# DIAGNOSTIC COMPETENCE IN BONE TUMORS: INFLUENCE OF ONCO-ORTHOPEDIC TRAINING

**DOI:** 10.1590/1413-785220253301e282483

**Published:** 2025-02-03

**Authors:** Julia Pozzetti Daou, Caio Falk Giannotti, Jairo Greco Garcia, Marcelo de Toledo Petrilli, Dan Carai Maia Viola, Reynaldo Jesus Garcia

**Affiliations:** 1 Universidade Federal de São Paulo, Department of Orthopaedics and Traumatology, Discipline of Orthopaedic Oncology, São Paulo, SP, Brazil; 2 Hospital Israelita Albert Einstein, Orthopedic Oncology Group, São Paulo, SP, Brazil

**Keywords:** Internship and Residency, Education, Medical, Neoplasms, Bone and Bones, Diagnostic Errors, Delayed Diagnosis, Internato e Residência, Educação Médica, Neoplasias, Osso e Ossos, Erros de Diagnóstico, Diagnóstico Tardio

## Abstract

Understanding the nature of musculoskeletal tumors is crucial for appropriate management and to secure a favorable prognosis. Orthopedists’ ability to identify these pathologies early and accurately is paramount. There is a requirement to raise awareness within the orthopedic community regarding the necessity of referrals to orthopedic oncology specialists, as delays in initiating proper treatment can compromise patient prognosis.

**Objective:**

The objective was to assess the capability of non-specialist orthopedists in identifying bone lesions suggestive of tumors and thus classify them by employing a questionnaire with radiographs and comparison with specialists. We aim to gain an in-depth understanding of their diagnostic competence and provide insights into teaching the subject in orthopedic residency programs.

**Methods:**

The sample consisted of 90 participants who answered the questionnaire: 18 orthopedic oncology specialists, 58 non-specialist orthopedists, and 14 orthopedic residents.

**Results:**

Specialists achieved an average accuracy of 12.50 ± 1.07, while non-specialists scored 10.00 ± 0.60 (p<0.001). Among non-specialists, there was no statistical significance when comparing whether they underwent specialization internship during residency nor the duration of the year of such training. The period since graduation also indicated no differences.

**Conclusion:**

This study highlights the importance of referring patients with suspected tumors to specialized orthopedists. ***Level of Evidence V; Expert Opinion.***

## Resumo

Compreender a natureza dos tumores musculoesqueléticos é crucial para a abordagem adequada e prognósticos favoráveis. A capacidade de identificação precoce e correta dessas patologias por ortopedistas é uma questão importante. Busca-se conscientizar a comunidade ortopédica sobre a necessidade de encaminhamentos para especialistas em oncologia ortopédica, uma vez que o atraso no início do tratamento adequado pode comprometer o prognóstico do paciente.

**Objetivo:**

O objetivo foi avaliar a capacidade de ortopedistas não especialistas em tumor musculoesquelético em identificar lesões ósseas sugestivas de tumores e classificá-las a partir de questionário com radiografias e da comparação com especialistas. Pretendemos obter uma visão aprofundada de sua competência diagnóstica, bem como traçar um panorama do ensino do tema nos serviços de residência médica.

**Métodos:**

A amostra consistiu em 90 participantes que responderam ao questionário eletrônico: 18 especialistas em onco-ortopedia, 58 ortopedistas não especialistas e 14 residentes em ortopedia.

**Resultados:**

Os especialistas tiveram média de acerto de 12,50 ± 1,07, enquanto os não especialistas, 10,00 ± 0,60 (p<0,001). Dentre os não especialistas, não houve significância estatística quando comparados se tiveram estágio na especialização durante a residência, nem quanto à duração do estágio ou em que ano da residência teve tal treinamento. O tempo de formado também não mostrou diferenças.

**Conclusão:**

O estudo aponta a importância de encaminhar pacientes com suspeita de tumores para ortopedistas especializados. **Nível de Evidência V; Opinião de Especialistas**

## INTRODUCTION

Primary malignant bone and soft tissue tumors, whilst considered rare, approximate 1% of malignancies, which pose significant challenges to orthopedic practice. To better understand the nature of these lesions proves crucial in determining the appropriate therapeutic approach and as such, achieve a more favorable prognosis. However, the ability for early and accurate detection of these pathologies by non-specialist orthopedic practitioners in orthopedic oncology remains an issue that is deserving of further attention.^1^,^2^,^3^

The inherent complexity in interpreting images of musculoskeletal tumors adds a layer of difficulty to the diagnostic approach. This challenge is particularly evident when considering the variety of tumors and pseudotumoral conditions that can emerge in radiological examinations which often present similar imaging characteristics. Within this context, radiography, being the initial exam, may not prove sufficient to accurately differentiate the nature of the lesions by a lesser trained individual. Failure to interpret imaging findings may indeed lead to inappropriate clinical management and treatment.^1^,^3^,^4^ The importance of this work transcends the mere assessment of the individual orthopedic competence. It aims to raise awareness among the orthopedic community as to the requirement for early referrals to orthopedic oncology specialists, as delays in initiating appropriate treatment, both for malignant and benign tumors, can significantly compromise patient prognosis.^5^

This article’s principal objective is to evaluate the ability of orthopedists who are not recognized specialists in musculoskeletal tumors to identify bone lesions suggestive of tumors and classify their nature based on radiographs via the application of a questionnaire and comparison with specialists’ responses. By assessing how these professionals approach the interpretation of imaging examinations and the classification of lesions as benign, malignant or aggressive benign, we set out to obtain an in-depth understanding of their diagnostic competence within this challenging context, and moreover, outline the landscape of teaching this subject in orthopedic residency programs.

## METHODS

This is a cross-sectional survey study approved by the Research Ethics Committee of the Universidade Federal de São Paulo (CEP-UNIFESP) and registered in Plataforma Brasil under the number 71176123.0.0000.5505/2023. The study was conducted at the Disciplina de Ortopedia Oncológica of the Departamento de Ortopedia e Traumatologia (DOT-UNIFESP).

The target population for this study consisted of orthopedic residents, orthopedic doctors specialized in oncology and orthopedic doctors without distinction based on subspecialization. Exclusion criteria were (A) refusal to participate in the study or (B) not in agreement with the content of the consent form.

The participants were requested to identify bone lesions suggestive of tumors and classify their nature based on radiographs via the application of a questionnaire. Due to the scarcity of similar studies within the Brazilian population - the restricted sample size, and the importance of evaluating the ability of orthopedists to identify potentially malignant lesions - a convenience sample was then selected.

**Figure 1:**
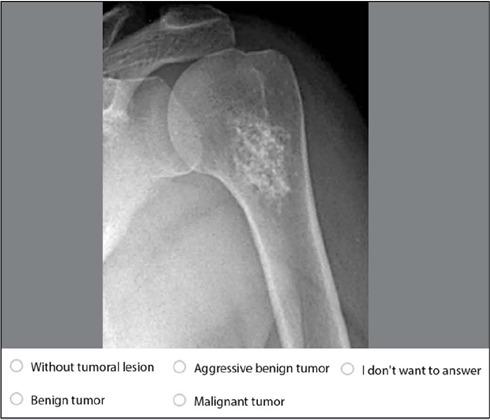
Example of a question to be answered by the participant in Google Forms.

The prepared questionnaire by researchers included sociodemographic data to characterize the sample and questions containing radiographic imagens and were required to indicate whether the radiographic images represented a tumor condition and to classify whether the lesion was malignant, benign, or aggressive benign (Figure 1).

**Figure 2:**
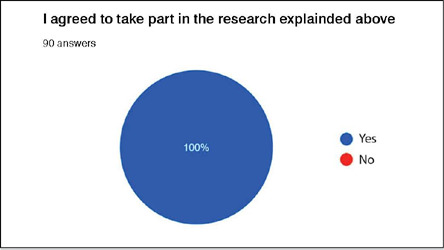
Proportion of participants who agreed to take part in the research and signed the Informed Consent Form.

The questionnaire was developed and reviewed within Google Forms. It was disseminated electronically on WhatsApp, in groups of orthopedists and residents via the following link: https://forms.gle/1akkWvtTBnoLgcrx8. The questionnaire was administered between November 2023 and January 2024. By agreeing to answer the questionnaire, the participant consented to participate in the study (Figure 2).

Quantitative variables were analyzed descriptively. Microsoft Excel software was applied for both descriptive and inferential analysis. Statistical analysis employed parametric tests using the software programs SPSS V26 (2019), Minitab 21.2 (2022), and Excel Office 2010, with a predetermined significance level of 5% (p<0.05) and an adjusted confidence interval (CI 95%).

## RESULTS

**Figure 3:**
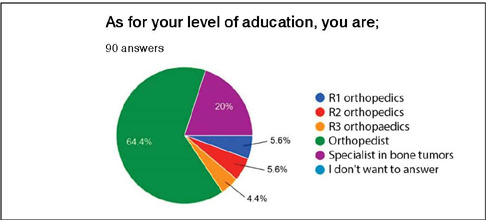
Characterization of the sample regarding the participants’ level of education (R1 = first year resident; R2 = second year resident; R3 = third year resident).

Our sample constituted 90 orthopedic surgeons, comprising 18 (20%) specialists in orthopedic oncology, 58 (64.4%) orthopedic oncology non-specialists, and 14 (15.6%) orthopedic resident physicians. Among the residents, there were five (5.6%) first-year residents (R1), five (5.6%) second-year residents (R2), and four (4.4%) third-year residents (R3) (Figure 3).”

**Figure 4:**
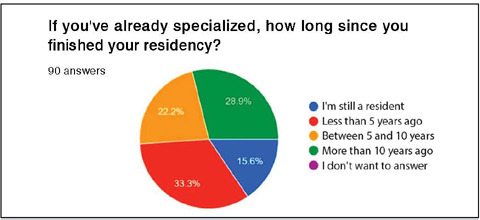
Characterization of the sample based on the completion time of orthopedic residency.

Among the 90 research participants, 26 (28.9%) completed their orthopedic training more than 10 years ago, 20 (22.2%) between 5 and 10 years ago, 30 (33.3%) within the last 5 years, and 14 (15.6%) were still residents at the time of the survey (Figure 4).

**Figure 5:**
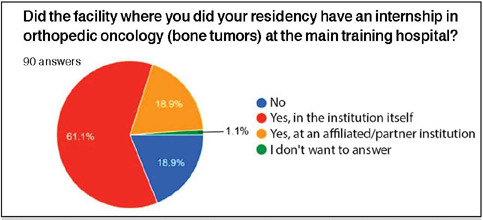
Proportion of participants who completed an internship in orthopedic oncology at the main hospital of their training.

As for training in orthopedic oncology, 55 (61.1%) participants underwent a specialty rotation at the main institution of their residency program, 17 (18.9%) at affiliated institutions of their primary center, 17 (18.9%) did not have this rotation in their training curriculum, and one (1.1%) participant did not respond to the question (Figure 5).

Of the 72 participants who underwent training in orthopedic oncology via the residency program, 51 (71%) completed this rotation for a period of 3 months or more, 16 (22%) for 1 to 2 months, and 5 (7%) for up to 1 month.

These 72 participants completed their internships as follows: 39 (54%) in only one year of residency, and 33 (46%) undertook the internship more than once throughout their term of residency (Figure 7A). Of these 33 participants, 26 (79%) underwent orthopedic oncology training in all three years of residency, two (6%) had training in the first two years of residency, one (3%) underwent the internship in the first and third years of residency, and four (12%) in the last two years of residency. Among the 39 participants who completed the internship only once during their term of residency, two (5%) did so only in the first year, seven (18%) only in the second year, and 30 (77%) only in the third year of residency (Figure 7B).

**Figure 6:**
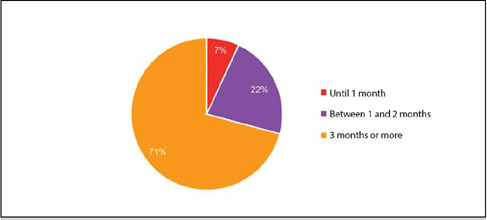
Characterization of the sample based on the duration of the orthopedic oncology rotation during residency.

**Figure 7:**
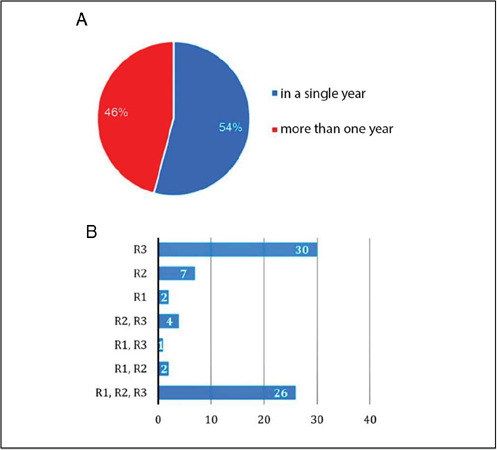
Participants who underwent the orthopedic oncology rotation multiple times. B. Distribution of the rotation during the residency years (R1 = first year resident; R2 = second year resident; R3 = third year resident).

**Figure 8:**
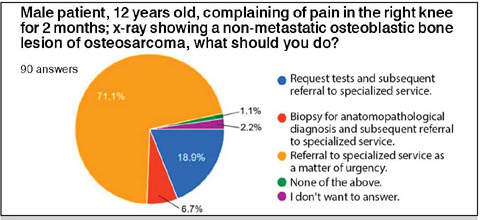
Participants’ responses regarding the approach to the presented clinical case.

Participants were presented with a clinical case of a 12-year-old child with suspected bone tumor and asked what course of action they would take. 64 (71.1%) participants would elect to refer to the specialized service without further tests; 17 (18.9%) would request tests before referring to the specialized service, six (6.7%) would perform a biopsy before referring to the specialized center, one (1.1%) would not do any of the alternatives proposed in the question and two (2.2%) did not wish to respond (Figure 8).

In relation to the identification of tumors, specialists recorded an average of 12.50 ± 1.07 correct answers, while non-specialists registered an average of 10.00 ± 0.60 questions (p<0.001) (Table 1). When comparing the results of questionnaires answered by non-specialists in relation to the time of completion of their orthopedic residency training, we found no statistically significant difference between the mean scores (Table 2).

**Table 1:** Comparison of mean accuracy between specialists and non-specialists in musculoskeletal tumors.

	Specialist	Non-Specialist
Mean	12.50	10.00
Median	13	10
Standard Deviation	2.31	2.58
CV	18%	26%
Min	7	6
Max	17	17
N	18	72
CI	1.07	0.60
P-value	<0.001	

**Table 2:** Comparison of mean accuracy among non-specialists in musculoskeletal tumors in relation to the time since completion of their orthopedic residency training.

	Resident	Less than 5 years	5-10 years	More than 10 years
Mean	9.43	10.36	8.93	10.93
Median	9	10	8	11
Standard Deviation	2.85	1.97	2.34	3.26
CV	30%	19%	26%	30%
Min	6	7	6	6
Max	16	14	13	17
N	14	28	15	15
CI	1.49	0.73	1.19	1.65
P-value	0.124			

There was no statistical significance among non-specialists when compared to duration of the internship during residency, as evidenced in Table 3.

**Table 3:** Comparison of mean accuracy among non-specialists in musculoskeletal tumors in relation to the duration of the internship during residency.

	Until 1 month	1 to 2 months	3 months or more
Mean	10.75	8.50	10.46
Median	10	8	10
Standard Deviation	2.36	2.50	2.57
CV	22%	29%	25%
Min	9	6	6
Max	14	13	17
N	4	12	41
CI	2.32	1.42	0.79
P-value	0.063		

When comparing whether non-specialists undertook an internship in oncologic orthopedics at the main institution, at a partner institution, or those who did not undergo internship, no significant statistical differences were observed, as shown in Table 4.

**Table 4:** Comparison of mean accuracy among non-specialists, stratified by the presence in the curriculum of the residency program.

	Não	Yes (Partnership)	Yes (Own)
Mean	9.73	9.91	10.11
Median	9	10	10
Standard Deviation	2.46	2.55	2.68
CV	25%	26%	26%
Min	6	6	6
Max	14	14	17
N	15	11	46
CI	1.25	1.51	0.77
P-value	0.883		

In relation to the time as to when participants underwent training in oncologic orthopedics, we divided the analysis into two parts: we first identified the last year they had contact with the subject and secondly, the number of years during residency in which internship was completed. None of the analyses yielded significant statistical results (Table 5).

**Table 5:** Comparison of mean accuracy according to the years of internship.

		Mean	Median	Standard Deviation	CV	Min	Max	N	CI	P-value
Last year of contact with the subject	R1	6.50	6.5	0.71	11%	6	7	2	0.98	0.129
	R2	9.71	10	2.81	29%	6	13	7	2.08	
	R3	10.27	10	2.57	25%	6	17	48	0.73	
Number of years they had the internship	1 year	9.86	9	2.81	29%	6	17	29	1.02	0.321
	2 years	8.80	10	1.64	19%	7	10	5	1.44	
	3 years	10.61	11	2.52	24%	6	16	23	1.03	

## DISCUSSION

This study comprehensively assessed the ability of non-specialist orthopedists in oncologic orthopedics to identify bone lesions suggestive of tumors and classify their nature based on radiographs by employing a questionnaire. Non-specialist responses were compared with those of specialists, which prompted statistical differences in just how these images were evaluated. Moreover, the study aimed to outline the landscape of teaching on this topic in orthopedic residency programs, given the scarcity of literature on the subject. Freeman T et al (2019) noted that out of the 11,773 articles published in the top 15 orthopedic journals in 2015, only 51 addressed education-related topics. The analysis of impact of formal oncologic orthopedic internships during residency - *on the diagnosis of bone lesions* - only further highlights the importance of specific experience in the field. It is pertinent to note that the Sociedade Brasileira de Ortopedis e Traumatologia corroborates the Resolution No. 22 of April 8, 2019, from the Ministry of Education, orthopedic residents should acquire the necessary competency to evaluate and manage the treatment of tumor lesions by the end of the third year of residency. That said, the lack of formal internships in oncologic orthopedics in various Brazilian residency programs underscores a gap in training, which could potentially contribute to the non-identification of tumor lesions in emergency or outpatient situations. It is therefore essential for all orthopedists to possess at least a basic understanding of musculoskeletal system tumors. This approach is crucial to optimize the effective use of time and resources, thus aiming for appropriate therapeutics within each clinical case.^5^,^6^,^7^ Undue delay in the diagnosis of bone tumors is problematic, given the potential decrease in the chances of successful treatment and increased morbidity after inadequate interventions. The two questions related to infections recorded the lowest accuracy rates, with one showing 1.1% correct responses, and the other, 5.5%. As infection is one of the principal differential diagnoses of bone tumors, it is crucial to highlight that in cases of diagnostic uncertainty, the most prudent approach is to refer the patient to a specialized oncologic service. This precaution aims to rule out the possibility of initiating treatment for infection when the underlying condition may, in reality, not be a tumor. Orthopedists should by right, be aware of the early referral of suspicious cases to specialists. For this reason, the referral question was established as the primary question guiding this study, which then revealed a rate of 71.1% favorable responses to the early referral approach, thus avoiding errors and diagnostic delays that can negatively influence the patient’s clinical outcome.

The first step in following patient cases with suspected bone tumors is the indication for biopsy. It is crucial to distinguish lesions that do not require this procedure, as some tumors exhibit unequivocal characteristics of benignity, avoiding the need for a biopsy. Furthermore, in cases where biopsy results do not alter the course of action, the performance of this procedure can be avoided. When biopsy is indicated, it is preferable that it be performed by a specialized team, guided by imaging exams to obtain tumor material in the best topographies and via the best access route for possible definitive surgery. Early diagnosis is crucial to maintaining the quality of life of patients, especially in the presence of metastases and skeletal events. In relation to soft tissue tumors, improper treatment results in compromised margins in 91% of patients and recurrence in at least 39%. It is essential to emphasize the importance of accurate diagnosis and appropriate treatment to optimize clinical outcomes and the patients quality of life.^8^,^9^,^10^,^11^

The limitations of this research include recognition that the extensive questionnaire, with 21 questions, may have potentially caused difficulties among participants; this, as well as the low number of orthopedists who responded to the questionnaire may have influenced the result. It was not assessed whether the orthopedist who answered the questions is associated with a teaching and resident training service, which could generate greater exposure to cases of musculoskeletal tumors. Additionally, it is important to note that radiography, despite being the exam of choice and in general, proving sufficient to form most diagnostic hypotheses, may indeed not be sufficient to identify a possible neoplastic lesion, especially in anatomically complex regions such as the pelvis. and spine. As such, MRI and CT scans can provide more detailed information. The choice to use solely radiographs in this study is due to their greater accessibility, as they are often the first point of approach to identifying injuries in an emergency care context.

Whilst this study highlights that radiography is not the most detailed of examination, it did prove sufficient for specialists in musculoskeletal tumors to identify, on average, more than half of the nature of the presented lesions (12,50 ± 1,07). Non-specialists however, did not achieve the same results (10,00 ± 0,60), which only emphasized the importance of prompt referral to specialized services.

Furthermore, the study indicated that the duration of time since completing orthopedic training, for the non-specialists in oncologic orthopedics, does not influence discriminatory capacity. This likely occurs due to the rarity of musculoskeletal tumors, which results in sporadic experience with these cases, thus the lack of generating diagnostic experience for the non-specialist.

No significant differences were identified between whether or not training in musculoskeletal tumors was conducted during the residency period. Despite the absence of differences in terms of duration of internship, a trend appeared in which participants with latter contact with the subject in the third year of residency scored more correct answers than those with last contact in their second and first years of residency, in that order. Participants who underwent specialization training in all three years also scored more correct answers. Within this context, various hypotheses could be considered: the annual completion of the specialist title exam by the Brazilian Society of Orthopedics, for which all residents of accredited services prepare, may contribute to uniformity in theoretical knowledge on the subject; or alternatively, questions may be raised in relation to the onco-orthopedic teaching offered in residency programs.

## CONCLUSION

When compared with recognized specialists, the ability of non-specialist orthopedists in identifying bone lesions suggestive of tumors and then classifying their nature based on radiographs proved to be statistically lower. However, various training modalities during medical residency for non-specialists, as well as time duration since completion of training, failed to unearth any significant statistical difference.

The results obtained can be directed as guidance for non-specialist orthopedists by emphasizing the importance of promptly referring patients with suspected musculoskeletal tumors to specialized orthopedists, and the requirement to more diligently evaluate the training of residents, as this can pose a direct influence on the outcomes of patient treatment and prognosis.
